# Multiplex Label Free Characterization of Cancer Cell Lines Using Surface Plasmon Resonance Imaging

**DOI:** 10.3390/bios9020070

**Published:** 2019-05-27

**Authors:** Ivan Stojanović, Carolina F. Ruivo, Thomas J. G. van der Velden, Richard B. M. Schasfoort, Leon W. M. M. Terstappen

**Affiliations:** 1 Medical Cell BioPhysics Group, MIRA institute, Faculty of Science and Technology, University of Twente, P.O. Box 217, 7500AE Enschede, The Netherlands; ivanstojanovic172@msn.com (I.S.); cruivo@ipatimup.pt (C.F.R.); r.b.m.schasfoort@utwente.nl (R.B.M.S.); 2 IBIS Technologies B.V., Pantheon 5, 7521PR Enschede, The Netherlands; Thomas@ibis-spr.nl

**Keywords:** Surface Plasmon Resonance imaging, Surface Plasmon Resonance imaging cytometry, flow cytometry, multiplex, cancer cell lines, label free

## Abstract

Rapid multiplex cell surface marker analysis can expedite investigations in which large number of antigens need to be analyzed. Simultaneous analysis of multiple surface antigens at the same level of sensitivity is however limited in the current golden standard analysis method, flow cytometry. In this paper we introduce a surface plasmon resonance imaging (SPRi)-based technique for 44-plex parameter analysis using a single sample, in less than 20 min. We analyzed the expression on cells from five different cancer cell lines by SPRi on a 44-plex antibody array including 4 negative controls and compared the output with flow cytometry. The combined correlation of the markers that showed expression by flow cytometry was 0.76. The results demonstrate as a proof of principle that SPRi can be applied for rapid semi-quantitative multiplex cell surface marker analysis.

## 1. Introduction

Recently the desire for high throughput multiplex cell analysis has grown because diseases like cancer involve complex cell surface antigen expression patterns. Several techniques like fluorescent microscopy and flow cytometry (Fluorescence-Activated Cell Sorting or “FACS”) have proven to be useful for multiplex analysis, but they are time consuming and multiplexing is limited by the available stains and filters of the respective set up. A 19-parameter FACS setup was reported [1], but its difficulty of use, data interpretation and data presentation make the method unattractive. Though FACS remains the standard for cellular biomarker analysis, new applications of existing techniques are emerging. Multidimensional microscopic robot technology was introduced for high-throughput protein co-localization [2]. The authors describe a technique that cycles fluorescence tagging, imaging and bleaching in situ. The uniqueness of this technique is that it can map hundreds of different proteins in one sample as it visualizes molecular clusters as a so-called toponome map. Similarly, recently a 65-plex-cytometry biomarker platform was introduced (Zellkraftwerk GmbH, Leipzig, Germany). It combines microscopy with FACS-like data processing. Samples are loaded into a microfluidic chip in which they are fixated using paraformaldehyde. Then they are stained and bleached consecutively per requirement [3]. This makes the process lengthy and laborious. Alternative strategies for multiplexing with FACS were also published recently. Sukhdeo et al. showed a technique in which FACS and fluorescent cell barcoding of different cell samples is used to distinguish them in one sample and analyze them individually. Two different intracellular stains were used, whereas secondary antibodies that were used were all labeled with Alexa647 [4]. Multiplexing in this study refers to the simultaneous analysis of 1 marker in 3 different cell lines pooled into 1 sample.

FACS is almost unanimously seen as the gold standard for determination of antigen expression on cells. Some groups have focused on alternative techniques that are not based on FACS to eliminate some of the shortcomings related to using FACS for multiplex analysis. Optical dark field microscopy was combined with gold nanorod molecular probes (GNrMP) that were conjugated to antibodies instead of a fluorescent dye. Three markers were studied simultaneously, but the authors indicate that 15 or more are possible [5]. This technique addresses the shortcomings of FACS as the range of wavelength that can be used is limited. GNrMP works in between of 600–2000 nm, offering ample multiplexing capacity. Lee et al. have shown a surface-enhanced Raman scattering (SERS)-based cellular imaging technique that uses silica-encapsulated hollow gold nanospheres (SEHGNs). Three markers were analyzed and quantified on living cell samples simultaneously [6].

Here we propose, as a proof of principle, an alternative technique for multiplex cell analysis, surface plasmon resonance imaging (SPRi). Recently we reported the ability of SPRi to consistently detect EpCAM expression on various viable cancer cells, analyzing them in real time and label free [7]. Here we introduce SPRi for the simultaneous label free detection of 44 antigens on viable cells in less than 20 min. In addition, the ease of use of the system and the simple sample preparation is an improvement over more laborious and complex cell analysis alternatives. In our experiments flow cytometry was used as the reference technology for comparing the SPRi output. Antigen expression was quantified using QuantiBRITE^®^ PE beads and the relative expression ratio of each parameter for each cell line was compared with the Resonance Unit (RU) output of SPRi.

## 2. Materials and Methods

### 2.1. SPRi

For SPRi measurements IBIS MX96 was used (IBIS technologies B.V., Enschede, The Netherlands). The standard 100 µm flow cell height in the IBIS MX96 was replaced with a 300 µm one to optimize sample homogeneity in the flow cell upon injection of the cell sample. Additionally, it enables three times more cells to sediment on the sensor surface area of 120 mm^2^. The obtained homogenous cell sample injection and larger volume of culture medium improve the cell viability over time. Regions of interest (ROI’s) define the analysis and reference surface areas. Each analysis ROI had its own dedicated reference ROI, which was placed on a blank sensor surface area. After data collection the responses from the reference ROI’s were subtracted from each analysis ROI to obtain referenced output data. The ROI’s were 100 × 80 pixels (550 × 440 µm). The ROI’s signal is based on the average light intensity, or rather measuring the dip in reflected light over the surface of an ROI. A more detailed description of the SPR setup and the principles behind it can be found elsewhere [7,8]. In addition, a cross-sectional schematic view is provided in supplemental Figure S1.

### 2.2. CFM Spotter

For ligand immobilization on the sensor surfaces the Continuous Flow Microfluidic (CFM) spotter was used (Wasatch microfluidics LLC, Salt Lake City, UT, USA) [9]. Ligand immobilization buffer was used to prime the CFM system and to dilute the desired ligands. The immobilization protocol lasted 60 min. The CFM spotter can spot up to 48 ligands onto the sensor in a single run simultaneously under back and forth confined flow (or 96 ligands if double printing is used).

### 2.3. SPR Sensors

Easy2Spot^®^ pre-activated G-type Senseye^®^ sensors (Ssens bv, Enschede, The Netherlands) were used as SPR sensor surfaces. The sensors have a 100 nm hydrogel-like layer, which enables higher capacity coupling of ligands in the evanescent field and gives the ligands a level of mobility approaching in vivo-like circumstances. The sensors are pre-activated rendering EDC-NHS activation unnecessary.

### 2.4. Antibodies

Antibodies were selected based on expected differences in expression levels among the 5 cell lines. The antibodies that were used belong to three different categories providing information of tissue origin, recognizing antigens involved in cell adhesion and potential drug targets. Anti-human EpCAM/CD326, Her2 and EGFR antibody were provided by Immunicon Corp. (Huntingdon Valley, Philadelphia, USA). Anti-human CD3, CD8a, CD11c, CD14, CD19, CD20, CD25, CD33, CD45, CD56, CD61, CD66b, CD105, CD123, CD140a, CD146, CD235a, CD71, CD117, CD221, CD227, CD261, CD262, CD309, Her3, CD24, CD44, CD49a, CD49b, CD49c, CD49d, CD49e, CD49f, CD103, CD104, CD106, CD113, CD144, CD166, CD324, CD334 antibodies were purchased from Biolegend Inc. (San Diego, CA, USA). Anti-human CD113 was purchased from Santa Cruz Biotechnology Inc. (Santa Cruz, CA, USA). Anti-human CD103 was purchased from BD Biosciences (San Jose, CA, USA). Anti-human serum albumin (HSA) antibody was used as a negative control in the SPRi experiments and was purchased from Sigma-Aldrich Chemie GmbH (Steinheim, Germany). Anti-mouse IgG PE was purchased from Abcam (Cambridge, UK) and was used for staining of unlabeled antibodies in flow cytometry and as a negative control in the SPRi experiments.

### 2.5. Cells

The following cell lines were used: breast cancer cell lines MCF7 (ATCC HTB-22™) and SKBR3 (ATCC HTB-30™), the acute myelogenous leukemia cell line KG1a (ATCC CCL-246.1™), the osteosarcoma cell line MG-63 (ATCC CRL-1427™) and the large cell lung cancer cell line NCI-H460 (ATCC HTB-177™). The cells were cultivated in their appropriate complete culture medium and were harvested using trypsin (except KG1a), subsequently they were resuspended in complete culture medium to a concentration of 1 million cells/mL before injection as a cell sample in the SPRi apparatus. The flow cell volume was 36 µL, so without dilution ~36,000 cells sediment on the sensor area of 120 mm^2^. The dimensions of a spot with immobilized antibody was 500 × 830 µm^2^. So, on each spot, ~124 cells landed. The region of interest on each spot covered about 60% of a spot, so about 74 cells landed in the RoI and contributed to the SPRi signal.

### 2.6. Sensor Deactivation Agent

A 1% Bovine Serum Albumin solution (BSA) (Sigma-Aldrich Chemie GmbH, Steinheim, Germany) in sodium acetate immobilization buffer was used as a deactivation agent. A stock solution of 2-aminoethanol (MP Biomedicals LLC, Illkrich, France) was used to create a 100-mM 2-aminoethanol solution (pH 8) and used as an extra sensor deactivation step after the initial BSA deactivation.

### 2.7. Ligand Immobilization Buffer

A 10-mM solution of immobilization buffer (pH 4.5) was made using anhydrous sodium acetate (Sigma-Aldrich Chemie GmbH, Steinheim, Germany) and acetic acid (Merck Schuchardt OHG, Hohenbrunn, Germany). First, a 0.2-M stock solution was made of both components, then from these stock solutions 1.93 parts of sodium acetate were mixed with 3.07 parts of acetic acid, finally 95 parts of ultrapure demineralized water was added.

### 2.8. System Buffer

When viable cells were analyzed in the SPRi apparatus, the system buffer was their respective complete culture medium in order to prevent bulk shift differences.

### 2.9. Multiplex Cancer Cell Line Analysis Using SPRi

**Figure 1:**
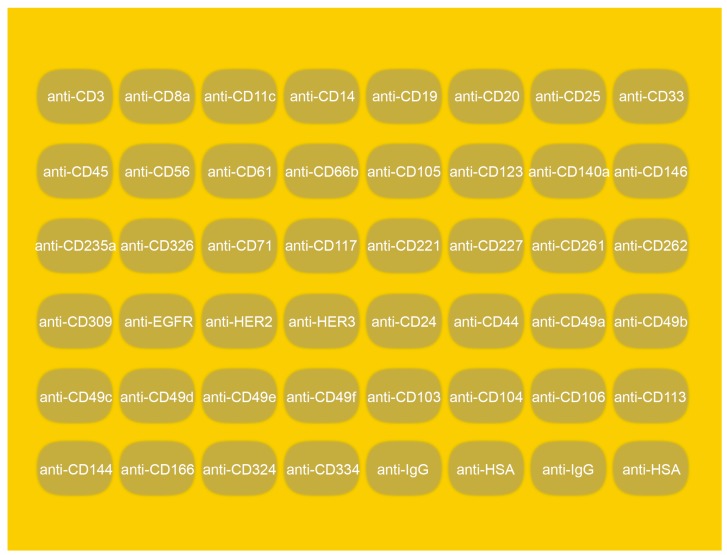
Schematic image of the 44 parameter Surface Plasmon Resonance imaging (SPRi) sensor. The 6 × 8 printhead created 48 spots with dimensions of 500 × 830 µm^2^ in a Surface Plasmon Resonance (SPR) image area of 6 × 8 mm.

The antibodies were diluted in ligand immobilization buffer to obtain a concentration of 5 µg/mL. Forty-eight wells of a 96-well plate were filled, of which 44 were filled with an anti-cell receptor antibody and four were used as negative controls (anti-HSA antibodies and anti-IgG antibodies). Figure 1 shows the layout of the antibodies on the sensor. The well plate and SPR sensor were then inserted in the CFM spotter and ligands were printed in 60 cycles of 1 min. After printing the sensor was inserted in the MX96 and sensor deactivation was performed with the 2 deactivation buffers (7 min each) [7]. After deactivation, the assay was programmed into the MX96. The cells are flowed over the sensor surface using a custom “cell analysis script” in which the sample plug containing the cells is flushed over the entire surface after which the flow is switched off, allowing the cells to sediment and interact with the ligands. An injection of 1 million cells/mL resulted in sedimentation of about 74 cells per RoI on a spot. Cells were allowed to interact for 900 s (15 min), after this association phase the back flow of fresh medium was restarted again.

### 2.10. Quantification of Cell Surface Markers by Flow Cytometry

To validate the data acquired by SPRi, a comparison was done using flow cytometry analysis. Experiments were done on a BD FACS Aria II^®^ flow cytometer (BD Biosciences, San Jose, CA, USA). The flow cytometer was equipped with 325, 488 and 633 nm wavelength lasers. Since the antibodies are unconjugated (for optimal SPRi performance), we had to use a secondary antibody (anti-IgG PE) to stain the antibodies for use in flow cytometry. This way the same antibodies from the same clone and batch could be used and performance of SPRi could be compared. The cells were prepared for flow cytometry using the following protocol. The cells were harvested and washed with PBS + 1% albumin (PBSA). The cells were centrifuged at 500 g for 10 min after which the pellet was resuspended to obtain 1 million cells/mL. One mL of this cell suspension was pipetted into a fluorescence-activated cell sorting (FACS) tube and then centrifuged for 10 min at 500 g. The pellet was resuspended in 50 µL of PBSA. Unconjugated antibody was added to reach a concentration of 1 µg/mL and incubated for 30 min at room temperature. Two washing steps with 2 mL PBSA and a subsequent 10 min at 500 g centrifugation were done. The pellet was resuspended in 50 µL of PBSA. PE conjugated anti-IgG antibody was added to reach an end concentration of 2 µg/mL, the sample was then incubated in the dark for 30 min at room temperature. The washing steps were repeated. The cell pellet was then fixated with a 1% formaldehyde solution for 10 min, after this the washing steps were repeated. Finally, the pellet was resuspended in 2-mL PBSA. Samples were made for all the cell lines separately with all the markers including a negative control sample containing just cells and the cells with just the anti-IgG PE antibody, this way a clear distinction could be made between background noise and a true positive signal. The expression levels of the separate markers on the different cell lines were quantified with the QuantiBRITE^®^ PE quantification kit (BD Biosciences, San Jose, CA, USA) using a previously published protocol [10].

### 2.11. Testing of Reproducibility

To determine the reproducibility of multiplex detection of cell surface marker expression on SPRi, we made a selection of markers for which we gathered 10 interaction results for each marker using 2 sensors. We have chosen for the repeats to be done on, SKBR3. We have chosen CD24 (high expression on SKBR3), CD49b (low expression), HER2 (high expression), EpCAM (mediocre expression) and CD8a (negligible expression) as markers to be analyzed for inter spot and inter sensor reproducibility and variability (% CV). After the assay the number of bound cells were counted on each of the ROI’s in order to be able to determine the CV percentage of number of cells.

In addition, we have analyzed the variability of cell sedimentation. For this a sensor was placed in the IBIS MX96 and cover coupled with a 25 µg/mL anti-HER2 solution dissolved in ligand immobilization buffer. The cell responses were recorded and analyzed for CV percentage.

## 3. Results

### 3.1. Multiplex Cancer Cell Line Analysis Using SPRi

**Figure 2:**
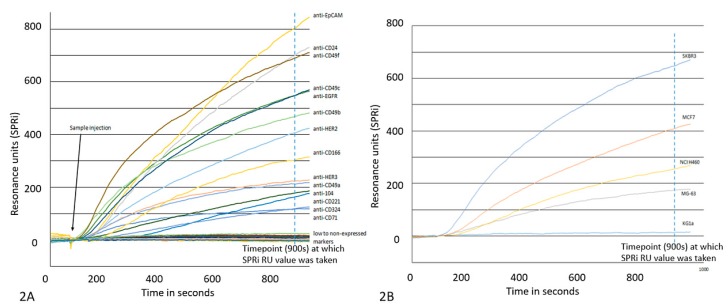
(**A**). Graphical output of MCF7 (ATCC HTB-22™) cells analyzed with SPRi. Shown are 44 markers which are expressed in varying levels on the cell surface of MCF7 cells. The higher the expression, the higher the response in RU. (**B**). Graphical output of a single marker (HER2) analyzed with SPRi across five different cell lines. Clearly visible is the difference in expression of the marker across the different cell lines, SKBR3 (ATCC HTB-30™) having the highest expression and KG1a (ATCC CCL-246.1™) the lowest.

**Figure 3:**
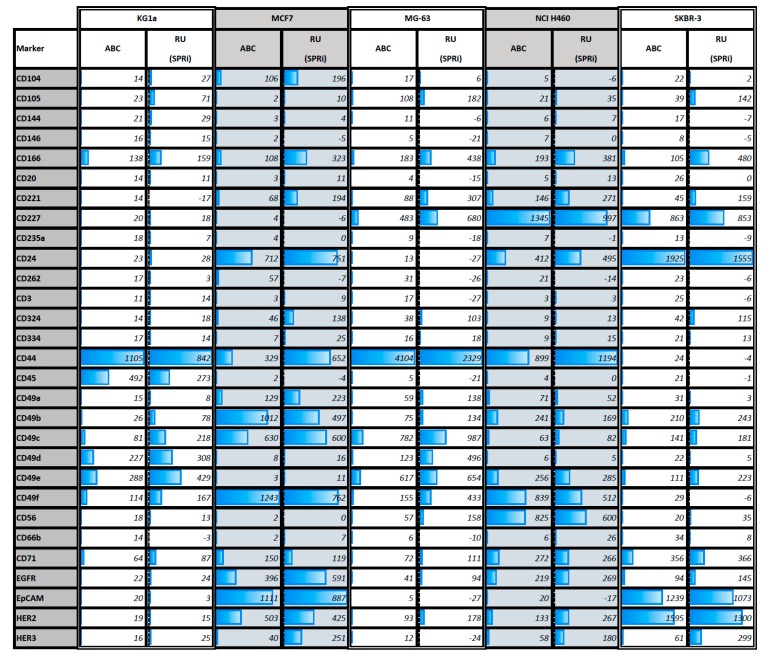
Graphical representation of the result table. The flow cytometric Antibody Bound per Cell (ABC) values and Resonance Units (RU) values of SPRi of five different cell lines are shown for 28 markers. In total, 16 markers were left out because these markers did not show any or a very weak response for all five cell lines (for the full unedited table including correlations see Supplemental Table S1). The total SPR signal per column was used to normalize the ABC signal with the SPR signal. It shows that all cell lines are different but some lines are “closer” to each other. E.g., the cell line KG1a and MG63 (ATCC CRL-1427™) behave similar over the various markers but are different with respect to CD45.

Figure 2A shows a SPR sensorgram illustrating the expression of all tested markers on cells from the breast cancer cell line MCF7. Figure 2B shows a SPR sensorgram of the expression of HER2 on the five different cell lines. The sensorgrams show a great divergence of expression on MCF7 cells and likewise different levels of HER2 expression on the five cell lines are seen. The higher the SPR response, the higher the cell surface expression of the antigen. The SPR RU endpoint values were recorded after 900 s as indicated with a dashed line in Figure 2A. This so-called cell response time (CRT) 900 value obtained from each cell line and each antibody are shown in Figure 3. Some markers also show negative SPR responses. The ABC values were normalized to the SPRi values in such a way that all ABC values were divided by the highest ABC value times the highest SPR value. The SPRi and ABC values were in addition bar plotted in Figure 3. We use the values obtained from the anti-IgG spots as a cutoff between positive and negative as the same anti-IgG PE antibody was used for the IgG PE control stain.

### 3.2. Quantification of Cell Surface Markers by Flow Cytometry

**Figure 4:**
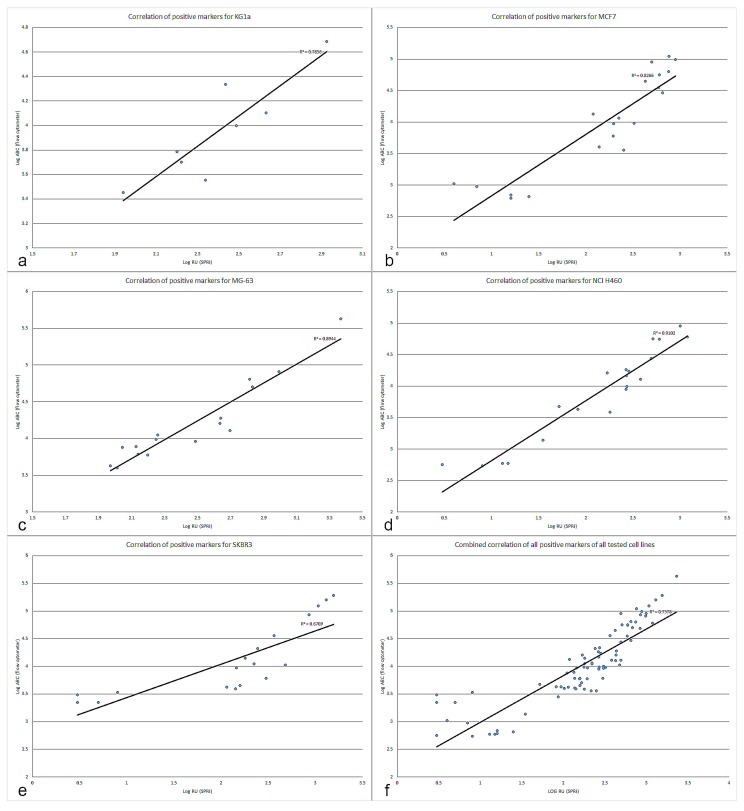
(**a**): Correlation plot of the positive markers for KG1a. (**b**): Correlation plot of the positive markers for MCF7. (**c**): Correlation plot of the positive markers for MG-63. (**d**): Correlation plot of the positive markers for NCI H460 (ATCC HTB-177™). (**e**): Correlation plot of the positive markers for SKBR3. (**f**): Combined correlation plot of all the positive markers of all the cell lines.

The geometrical mean values obtained with flow cytometry were used to determine the ABC value using the QuantiBRITE PE method. The ABC values represent the amount of antibodies that have been bound to a cell and the higher the value the higher the expression of the respective cell surface marker. The ABC output of flow cytometry and the CRT900 value of SPRi for each of the antibodies for the five cell lines were plotted against each other and shown in Figure 4a–f. For the correlation determination, only the markers with positive expression were used. The highest correlation was found for NCI H460 (R^2^ = 0.91) and the lowest correlation for SKBR3 (R^2^ = 0.67). Combined correlation was R^2^ = 0.76 (see Supplemental Table S1). ABC values for each cell line that are below the level of the unstained control have a white bar plot with a “low” value. The low values between the unstained and IgG PE control sample indicate very low or non-existing expression. Whereas all markers that had recorded values higher than the IgG PE control sample show a gradually increasing blue bar plot and a “high” value as their expression increased. The same was done with the CRT 900 value of SPRi (see Figure 3).

### 3.3. Testing of Reproducibility

The results of the reproducibility tests show that the SPRi technique is consistent in detecting approximate levels of expression, however there is also a considerable coefficient of variation (see Table 1). The positive markers never exceed a CV percentage of 33% (HER2) for the RU values, with the lowest CV value being that of EpCAM and CD49b (19%). When determining the CV values of the number of cells counted within the ROI of the respective marker the CV values stay within a similar range, however the maximum CV percentage is higher at 40% (HER2) and it is at 18% for the lowest values (CD24 and CD49b). CD8a (negligible expression) shows a much higher CV value of 187% for RU values when values are not corrected for the number of cells and a “normal” CV value of 30% for counted cells.

The experiment with the cover coupled sensor (see Supplemental Table S2) shows that despite a homogenously deposited antibody layer there is still a large variation in the number of cells binding (RU CV of 71% and cell count CV of 37%). Visual inspection of the sedimentation pattern shows that there are depletion zones visible that have fewer cells bound to the surface. These zones were located immediately next to the “O-ring” of the flow cell and right next to the middle of the flow cell, where both above and under the center flow lower amounts of cells were visible.

## 4. Discussion

Previously we reported that SPRi can be used to detect cell binding based on their cell surface marker expression [7]. Here we show that the SPRi platform is also suited for multiplex detection of cell surface markers. After sedimentation, the sensor response is continuously increasing. Our explanation is that cells which landed (and are exposed to the hydrogel surface) will interact with multiple anchoring points to the immobilized ligands. The higher number of cell surface antigens the more interactions will be formed with their respective ligands and the cells will both spread and interact deeper into the hydrogel (and therewith the evanescent field). Hence, the faster the SPRi response divided by the number of cells per ROI, the higher the expression of surface markers on the cell. The signal (in RU) is dependent on the number of cells that sediment in the ROI and the size of the ROI. The software only determines the SPR shift within the ROI. When a ROI is made smaller a sedimenting cell (provided it lands within the ROI) will give rise to a higher response. Consequentially when smaller regions of interest are used (for instance when a ROI would be made as small as a single cell) the system also becomes much more sensitive to noise. The main difference between both techniques is that in flow cytometry the antibodies are in solution and thus free to diffuse whereas in SPRi cytometry the antibodies are “immobilized” on the sensor surface. Additionally, SPRi measures only the interaction in an ROI of one side of the cell that penetrated in the evanescent field of the sensor surface. In flow cytometry, the antibodies can diffuse to their target without restrictions. In SPRi cytometry, the antibodies are coupled to the hydrogel. The worse mobility or too low ligand density of some of the immobilized antibodies could explain in some cases the lower levels of correlation between the two techniques. For example, CD262 (MCF7, MG-63, NCI-H460 and SKBR3) shows no expression using SPRi cytometry while flow cytometry showed a slight expression. The results in Figure 3 and the correlation plots in Figure 4a–f demonstrate that antigen expression density is contained within the SPRi responses and correlates well with flow cytometry. The ABC values show the different amounts of cell surface antigens on the tested cell lines. In Figure 3 the actual measured values are given but not the normalized values in order to show clear clean signals. The higher expressed markers are highlighted with a blue bar box plot, whereas the lower expressed markers have a narrower bar blue box plot. The same was done with the recorded SPRi responses. The general trend is comparable between the two techniques, the higher expressed markers (such as for instance CD44 and CD45 for KG1a; CD49f, EpCAM, CD49b, CD24, CD49c and HER2 for MCF7; CD44, CD49c, CD49e and CD227 for MG-63; CD227, CD44, CD56 and CD24 for NCI H460; CD24, HER2, EpCAM and CD227 for SKBR3) are correlating very well, whereas the low to intermediately expressed markers show a bit more deviation in their correlation, as can be seen in Figure 4f. Particularly SKBR3 shows a relatively high deviation in the correlation of the data. This can be explained by a lack of some SPRi markers in the analysis of the correlation. These markers (including CD261, CD140a and CD49f) were giving negative SPRi responses, even though they were low positive in flow cytometry. In future experiments special attention should be paid to the immobilization of these antibody spots and/or reducing the non-specific adhesion.

The SPRi data seems to show a deviation that leans towards higher positivity of the markers compared to the flow cytometry determined expression, it therefore seems that the low to medium expressed markers are better detectable using SPRi. This could be due to the fact that lower expressed markers are more difficult to quantify with flow cytometry and are close to the noise level of the instrument, considering the relative high background signal detected from the IgG PE control in some of the cell lines (such as KG1a, MG63 and SKBR3).

The main advantage of using SPRi for analyzing cell surface markers is the fact that all cells are analyzed simultaneously and can be analyzed within minutes of harvesting. No fixation is needed as the cells remain alive throughout the entire analysis, which is not the case in flow cytometry. The time needed to obtain results is also in favor of SPRi. A total assay takes only approximately 20 min, while full sensor preparation and obtaining sensorgrams takes approximately 1 h and 45 min. Obtaining 44 parameter results using flow cytometry would take an operator with average skills much longer.

A point of attention of quantifying cell surface marker expression using SPRi is that the IBIS MX96 instrument isn’t capable to perform the analysis at the single cell level, as the SPRi signals are measured in the ROI over the complete ligand covered spot in a fixed concentration of a million cells per mL. Improvement in the imaging to locate the exact position of the cells and software to only report the SPRi signals obtained from the cell locations can overcome these limitations. After injection of the cell sample cells sediment on the entire sensor surface. This means that the cells that land on the part of the sensor surface that is not covered by a ligand spot are wasted.

A much simpler way of performing single cell analysis would be recording the pixel data of the camera and subsequently analyzing only the parts of the sensor on which a single cell event has occurred. This would enable single cell analysis in which the sensorgrams would probably be more comparable to each other. In addition, this way of data recording and analysis enables the reuse of a sensor for several injections. Regeneration of the sensor would not be needed as with even the currently used concentrations of cells there is still plenty of space left on the ligand spots for concatenated single cell binding events to take place. As indicated the flow cell has a height of 300 micron and cells are injected simultaneously. However for each cell the sedimentation can vary between 40 to 80 s depending on the sedimentation time of the individual cell [11]. Hence the specific interaction time for a single cell to the sensor surface can vary between 820 and 900 s.

The quality of the sensorgram of cells that sediment and bind to the sensor surface is worse than compared to a protein-protein assay. This is due to the limited number of cells that cause a signal and the interaction process that differs for proteins (association and dissociation) and cells (binding and penetrating). Reproducibility of SPRi cytometry is also an important factor that needs to be addressed. In one of our previously published experiments, in which we pioneered SPRi cytometry using a similar method we did focus on reproducibility [7] and the results of 3 different cancer cell lines and their expression of the EpCAM cell surface marker are provided in Supplemental Table S3. The combined CV was determined to be 21%. For this paper, we have chosen a set of five different markers expressed on SKBR3 cells to test reproducibility. These were CD24 (high expression on SKBR3), CD49b (low expression), HER2 (high expression), EpCAM (mediocre to high expression) and CD8a (negligible expression). The resulting CV values (see Table 1) show that the variation are within the same range as previously published and therefore we can claim that the current way we perform SPRi cytometry has an inherent CV percentage of approximately 20–25%. We have also determined the RU/cell value for each of the markers (see Table 1 and Supplemental Figure S2).

The linearity of the SPR-dip shift and the response detected by the IBIS MX96 is of great importance for the semi-quantitative analysis of cell surface markers. Standard fixed angle SPRi applies the change in reflectivity which is not correlated to the effective dip shift and mass change. The IBIS MX96 applies the scanning angle principle [12], and all spots/ROI are calibrated to each other for correct refractive index shift measurements. The sensitivity of all spots is in this way exactly the same for all ROIs.

It is evident that the current way of analyzing cells using SPRi is not free of variation. The CV values of the RU/cell column and Supplemental Figure S2 are better and show that the response per cell is relatively consistent with values ranging between 8 and 19% for the positive markers. The CV’s in general can be explained in part by the fact that there is no control over the lateral distribution of cells that sediment toward the surface and interact with the immobilized ligands (See Supplemental Table S2). A CV percentage of 71% for the RU value was determined among the ROI’s, if cell injection would be more homogenous and not influenced by flow patterns this CV value would improve. Low ligand densities can be spotted and it was observed that even these low values can give high RU responses. Supplemental Figure S3 shows the responses of the cell line HS578T (originating from a carcinosarcoma). The sensorgram shows that the highest responses caused by this cell line are seen on the lowest ligand density CD44 spot, due to the fact that CD44 is highly expressed by these cells. This shows that ligand density does not determine, but it is the cell expression of a cell surface marker that is the major determinant of a SPRi response.

One aspect to note is that the sensor used for supplemental Figure S4 was regenerated with a short burst of 1%-TritonX solution. This “dissolved” any bound cells on the sensor surface, but it is not capable to properly regenerate the surface from any bound proteins in addition to the fact that TritonX denatures proteins (and therefore also the antibodies used as ligands). Consequentially, the sensor surface would become less reactive after each regeneration cycle and therefore the recorded RU’s were lower than the ones recorded in the main data set seen in Figure 3, for which a new sensor was used for each analysis.

Additionally, the SPRi output is given in the value RU which is a correlated value for the number of surface markers whereas flow cytometry can be calibrated by for example QuantiBRITE^®^ beads to arrive at an absolute defined number of surface markers expressed by each cell. Such standards should in principle also be made for SPRi, because the data correlate with each other. In comparison studies the SPR data can be calibrated to the quantitative ABC flow cytometry data. In cancer treatment research, more value is given to the cell surface ratios of the antigens rather than exact numbers of cell surface antigens. This means that any highly expressed marker could be potentially of interest, their exact number of expressions is less relevant. Though having 44 markers analyzed simultaneously is already a very high number, it is conceivable that in the future even higher levels of multiplexity might be required. For this our proposed method can be easily expanded to accommodate this desire.

We believe that SPRi offers unique advantages that can supplement flow cytometry with regards to cell surface marker analysis. It is a relatively simple technique that is easy to learn and operate. It applies comparatively inexpensive unlabeled antibodies and live cells can be injected and tracked in real-time. It offers high multiplex power and detection in a very short time frame. Its output gives a good impression of the ratios of cell surface markers present on a cell surface.

## 5. Conclusions

In this proof of principle paper, we have shown that SPRi is a suitable technique for rapid multiplex analysis of cell surface marker expression levels. The SPRi signal is proportional to the anchoring points of ligand–cell surface antigens interactions generating more cell spread. Additionally, the cell penetrates deeper in the hydrogel of the sensor surface that give rise to higher signals. We analyzed KG1a, MCF7, MG63, NCI H460 and SKBR3 cells for their expression levels of 44 cell surface markers. Data acquired using SPRi correlates well with the golden standard flow cytometry based technique for cell surface marker quantification (QuantiBRITE^®^). The determined correlations (R^2^) of all positive markers in order from highest to lowest were: 0.91 (NCI H460), 0.89 (MG63), 0.82 (MCF7), 0.79 (KG1a) and 0.67 (SKBR3). Combined correlation of all positive markers across all cell lines was determined to be 0.76. The data shows that SPRi is not only capable of analyzing cell surface marker expression on living cells in multiplex fashion, but to our knowledge, for the first time we show that SPRi output also contains information about the expression ratios of these cell surface markers. SPRi cytometry is able to provide the data in less than 20 min (analysis time) as opposed to measuring the markers sequentially in series as is done usually in flow cytometry, which would take much longer.

## Figures and Tables

**Table 1:** Responses of the reproducibility test for CD24, CD49b, HER2, EpCAM and CD8A.

Run#	CD24 (RU)	# of Cells	RU/Cell	CD49b (RU)	# of Cells	RU/Cell	HER2 (RU)	# of Cells	RU/Cell	EpCAM (RU)	# of Cells	RU/Cell	CD8a (RU)	# Of Cells	RU/Cell
1	554	20	28	218	15	15	968	29	33	386	15	26	−32	10	−3
2	462	20	23	205	12	17	1083	31	35	465	21	22	−36	16	−2
3	533	22	24	161	12	13	633	23	28	399	17	23	−32	10	−3
4	298	16	19	301	19	16	725	26	28	530	22	24	87	23	4
5	379	18	21	271	18	15	676	26	26	339	18	19	27	20	1
6	299	17	18	226	14	16	553	21	26	591	23	26	48	21	2
7	338	24	14	230	16	14	1279	55	23	517	22	23	35	20	2
8	359	17	21	178	12	15	612	23	27	543	24	23	178	28	6
9	239	13	18	266	18	15	1140	52	22	616	29	21	27	19	1
10	419	22	19	200	15	13	554	22	25	582	25	23	42	22	2
St. dev.	104	3	4	43	3	1	270	12	4	95	4	2	65	6	3
Average	388	19	20	226	15	15	822	31	27	497	22	23	34	19	1
CV (%)	27	18	19	19	18	8	33	40	15	19	19	9	187	30	299

## Supplementary Materials

The following are available online at https://www.mdpi.com/2079-6374/9/2/70/s1, Table S1: Responses of all 44 markers obtained with flow cytometry (ABC) and SPRi cytometry (RU SPRi), the data is sorted from high expression (green colors) to low expression (red colors) (as determined by flow cytometry), Table S2: Variation of cell binding seen on a cover coupled sensor, Table S3: Reproducibility values of 3 different cancer cell lines expressing the EpCAM cell surface maker in different amounts, Figure S1: Cross-sectional schematic of the used SPRi apparatus, Figure S2: Graph plotting the RU/cell responses of all the markers and repeat experiments performed, Figure S3: Sensorgram showing HS578T cells expressing a very high amount of CD44, despite the fact that the CD44 antibodies were immobilized with the lowest concentration of all the used ligands, Figure S4: Overlay plot showing different amounts of MCF7 cells being analyzed for EGFR expression. As the cell concentration decreases approximately by half, so does the SPRi response.
