# Supplementary material for: Multiplex Label Free Characterization of Cancer Cell Lines Using Surface Plasmon Resonance Imaging
Source: Biosensors (Basel). 2019 May 27;9(2):70. doi: 10.3390/bios9020070 (PMC6628007; doi:10.3390/bios9020070)
Supplement: Supplementary file 1 [file biosensors-09-00070-s001.pdf]

Supplementary Materials

Article

Multiplex Label Free Characterization of Cancer Cell Lines Using Surface Plasmon Resonance Imaging

Ivan Stojanović [1], Carolina F. Ruivo [1], Thomas J.G. van der Velden [2], Richard B.M. Schasfoort [1] and Leon W.M.M. Terstappen [1,*]

[1] Medical Cell BioPhysics Group, MIRA institute, Faculty of Science and Technology, University of Twente, P.O. Box 217, 7500AE Enschede, The Netherlands; ivanstojanovic172@msn.com (I.S.); cruivo@ipatimup.pt (C.F.R.); r.b.m.schasfoort@utwente.nl (R.B.M.S.)

[2] IBIS Technologies B.V., Pantheon 5, 7521PR Enschede, The Netherlands; Thomas@ibis-spr.nl

* Correspondence: l.w.m.m.terstappen@utwente.nl; Tel.: +31-53-489-2425

Received: 28 April 2019; Accepted: 22 May 2019; Published: 27 May 2019

Table S1. responses of all 44 markers obtained with flow cytometry (ABC) and SPRi cytometry (RU SPRi), the data is sorted from high expression (green colors) to low expression (red colors) (as determined by flow cytometry).

KG1a			MCF7			MG-63			NCI-H460			SKBR3		
**Marker**	**ABC**	**RU (SPRi)**	**Marker**	**ABC**	**RU (SPRi)**	**Marker**	**ABC**	**RU (SPRi)**	**Marker**	**ABC**	**RU (SPRi)**	**Marker**	**ABC**	**RU (SPRi)**
CD44	48639	842	*CD49f*	110012	762	*CD44*	426853	2329	*CD227*	89832	997	*CD24*	191709	1555
CD45	21659	273	*EpCAM*	98284	887	*CD49c*	81333	987	*CD44*	60073	1194	*HER2*	158838	1300
CD49e	12663	429	*CD49b*	89604	497	*CD49e*	64212	654	*CD49f*	56021	512	*EpCAM*	123384	1073
CD49d	9994	308	*CD24*	62994	751	*CD227*	50185	680	*CD56*	55119	600	*CD227*	85931	853
CD166	6090	159	*CD49c*	55734	600	*CD166*	19032	438	*CD24*	27534	495	*CD71*	35500	366
CD49f	5015	167	*HER2*	44525	425	*CD49f*	16082	433	*CD71*	18187	266	*CD49b*	20942	243
CD49c	3580	218	*EGFR*	35005	591	*CD49d*	12793	496	*CD49e*	17089	285	*CD49c*	14023	181
CD71	2822	87	*CD44*	29102	652	*CD105*	11200	182	*CD49b*	16082	169	*CD49e*	11037	223
CD123	1260	40	*CD71*	13296	119	*HER2*	9684	178	*EGFR*	14629	269	*CD166*	10486	480
CD49b	1141	78	*CD49a*	11458	223	*CD221*	9135	307	*CD166*	12875	381	*EGFR*	9325	145
CD61	1039	10	*CD166*	9564	323	*CD49b*	7787	134	*CD221*	9766	271	*HER3*	6050	299
CD24	1017	28	*CD104*	9419	196	*CD71*	7531	111	*HER2*	8873	267	*CD221*	4499	159
CD105	1013	71	*CD221*	5994	194	*CD49a*	6100	138	*CD49a*	4731	52	*CD324*	4206	115
CD33	959	2	*CD262*	5075	-7	*CD56*	5928	158	*CD49c*	4241	82	*CD105*	3903	142
EGFR	957	24	*CD324*	4032	138	*EGFR*	4261	94	*HER3*	3858	180	*CD66b*	3386	8
CD261	957	7	*HER3*	3584	251	*CD324*	3980	103	*CD262*	1396	-14	*CD49a*	3046	3
CD144	924	29	*CD261*	1052	4	*CD262*	3183	-26	*CD105*	1371	35	*CD261*	3035	-9
CD227	901	18	*CD103*	948	7	*CD113*	1895	27	*EpCAM*	1365	-17	*CD140a*	2926	-10
EpCAM	860	3	*CD49d*	691	16	*CD104*	1754	6	*CD113*	1095	-8	*CD49f*	2904	-6
HER2	817	15	*CD113*	672	1	*CD3*	1732	-27	*CD324*	588	13	*CD14*	2679	-16
CD235a	802	7	*CD334*	657	25	*CD334*	1656	18	*CD334*	588	15	*CD20*	2548	0
CD56	794	13	*CD61*	613	16	*CD106*	1613	-29	*CD140a*	567	3	*CD3*	2510	-6
CD262	738	3	*CD11c*	379	4	*CD24*	1300	-27	*CD123*	541	8	*CD44*	2341	-4
CD140a	736	2	*CD235a*	348	0	*CD103*	1300	-15	*CD146*	478	0	*CD25*	2319	-5

CD334	732	14	*CD227*	339	-6	*HER3*	1251	-24	*CD117*	466	-1	*CD262*	2281	-6
CD103	725	15	*CD14*	268	-1	*CD144*	1187	-6	*CD235a*	435	-1	*CD103*	2237	3
HER3	715	25	*CD49e*	265	11	*CD117*	1095	-21	*CD144*	422	7	*CD49d*	2226	5
CD146	715	15	*CD144*	265	4	*CD11c*	1036	-24	*CD66b*	410	26	*CD61*	2210	6
CD11c	713	-4	*CD20*	247	11	*CD261*	1036	-30	*CD33*	408	-6	*CD106*	2210	-5
CD309	706	25	*CD106*	245	0	*CD309*	1015	-17	*CD61*	397	-5	*CD104*	2194	2
CD117	704	4	*CD3*	239	9	*CD235a*	972	-18	*CD106*	389	-1	*CD8a*	2188	-16
CD113	691	26	*CD117*	239	-2	*CD14*	801	-27	*CD19*	376	2	*CD45*	2134	-1
CD106	685	10	*CD8a*	235	-3	*CD8a*	790	-28	*CD49d*	374	5	*CD113*	2128	89
CD49a	679	8	*CD33*	229	-3	*CD66b*	614	-10	*CD104*	350	-6	*CD334*	2047	13
CD25	679	7	*CD19*	227	0	*CD45*	551	-21	*CD261*	331	9	*CD56*	1960	35
CD66b	634	-3	*CD25*	227	5	*EpCAM*	487	-27	*CD20*	308	13	*CD11c*	1895	-5
CD221	630	-17	*CD309*	227	6	*CD146*	487	-21	*CD103*	284	3	*CD19*	1862	-7
CD20	630	11	*CD56*	199	0	*CD123*	455	-17	*CD25*	280	6	*CD33*	1857	-14
CD104	617	27	*CD45*	178	-4	*CD25*	434	-17	*CD14*	272	-4	*CD123*	1732	5
CD324	600	18	*CD140a*	174	-6	*CD140a*	434	-35	*CD45*	270	0	*CD144*	1683	-7
CD8a	529	4	*CD146*	170	-5	*CD20*	413	-15	*CD11c*	261	-5	*CD117*	1678	-10
CD14	520	6	*CD123*	166	10	*CD33*	387	-10	*CD8a*	231	-12	*CD309*	1651	3
CD19	512	4	*CD105*	156	10	*CD19*	355	-27	*CD3*	223	3	*CD235a*	1319	-9
CD3	468	14	*CD66b*	137	7	*CD61*	334	-21	*CD309*	193	-6	*CD146*	779	-5
Correlation	0.7856		*Correlation*	0.8266		*Correlation*	0.8944		*Correlation*	0.9102		*Correlation*	0.6709	
Combined correlation	0.7578													
Unstained (flow cytometer)/anti HSA (SPRi)	153	19	*Unstained*	209	8	*Unstained*	1911	-13	*Unstained*	131	-20	*Unstained*	1373	1
IgG PE control	1280	4	*IgG PE control*	397	-2	*IgG PE control*	2453	-26	*IgG PE control*	424	-6	*IgG PE control*	2225	-6

Table S2. Variation of cell binding seen on a cover coupled sensor.

Roi nr	Amount of Cells	RU	RU/Cell
2	22	120	5
3	18	176	10
4	25	194	8
5	23	395	17
6	17	202	12
7	18	199	11
8	16	319	20
9	15	246	16
10	18	328	18
11	18	331	18
12	16	326	20
13	12	66	5
14	18	143	8
15	25	326	13
16	15	113	8
17	24	240	10
18	29	375	13
19	28	626	22
20	33	625	19
21	38	579	15
22	40	827	21
23	37	821	22
24	20	609	30
25	10	−28	-3
26	10	−56	-6
27	23	256	11
28	14	42	3
29	18	136	8
30	24	341	14
31	11	−34	−3
32	21	230	11
33	19	307	16
34	8	80	10
35	15	288	19
36	28	487	17
37	21	245	12
38	34	594	17
39	27	316	12
40	28	458	16
41	23	192	8
42	36	670	19

43	35	505	14
44	25	201	8
45	10	67	7
46	14	133	10
47	21	497	24
48	25	683	27
st. dev	8	221	19
average	22	311	16
CV (%)	37	71	121

Table 3. Reproducibility values of 3 different cancer cell lines.

Experiment	EpCAM on MFC7	EpCAM on SKBR3	EpCAM on HS578T
	Response in RU	Response in RU	Response in RU
1	1213	704	1898
2	1382	782	2242
3	1543	973	1860
4	1363	884	1938
5	1506	678	1886
6	1537	807	2152
7	1359	1061	1522
8	1946	910	1480
9	1167	1043	1649
10	1758	1269	1359
11	1733	925	1808
12	1403	1168	2299
13	1731	1589	2192
14	1607	1292	1645
15	1767	1166	1674
16	1787	1406	1622
17	1531	862	1522
18	1548	1040	1378
19	1698	907	1561
20	2249	1041	2282
21	1759	766	1544
22	2864	730	1658
23	1710	981	1349
24	1489	1297	1280
25	1699	1160	1221
26	1586	1083	1519
27	2252	1355	1572
28	2264	1233	1732

29	*1636*	*1157*	*1345*
30	*1751*	*989*	*1219*
Average	*1695*	*1042*	*1680*
Mean	*1667*	*1041*	*1634*
St. dev.	*348*	*225*	*317*
CV	*21*	*22*	*19*
Combined CV	*21*		

Supplemental expressing the EpCAM cell surface maker in different amounts.

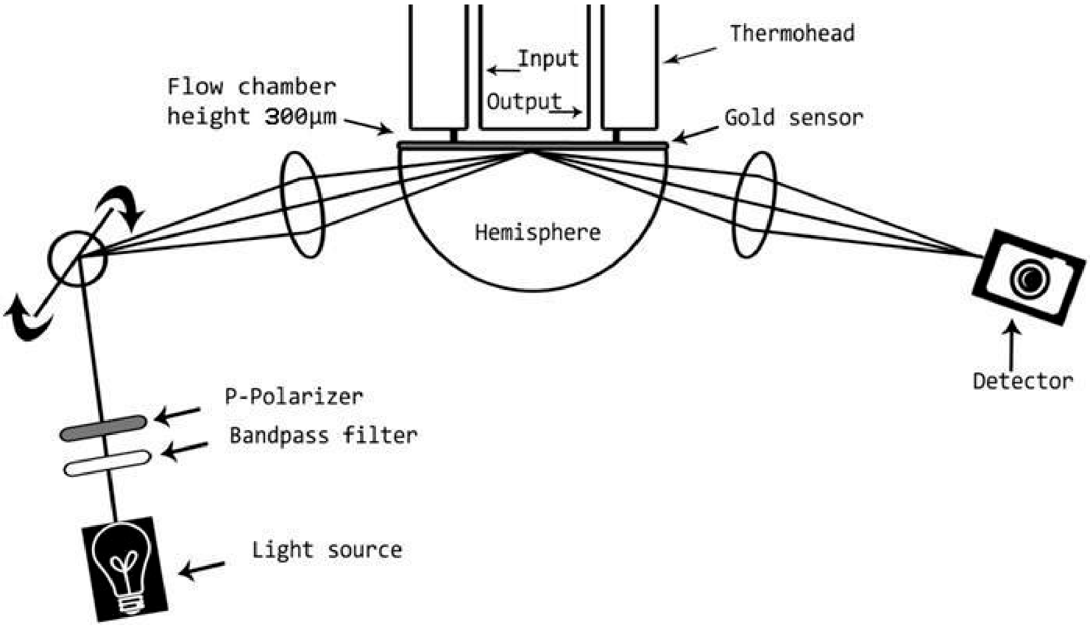

Figure S1. Cross-sectional schematic of the used SPRi apparatus.

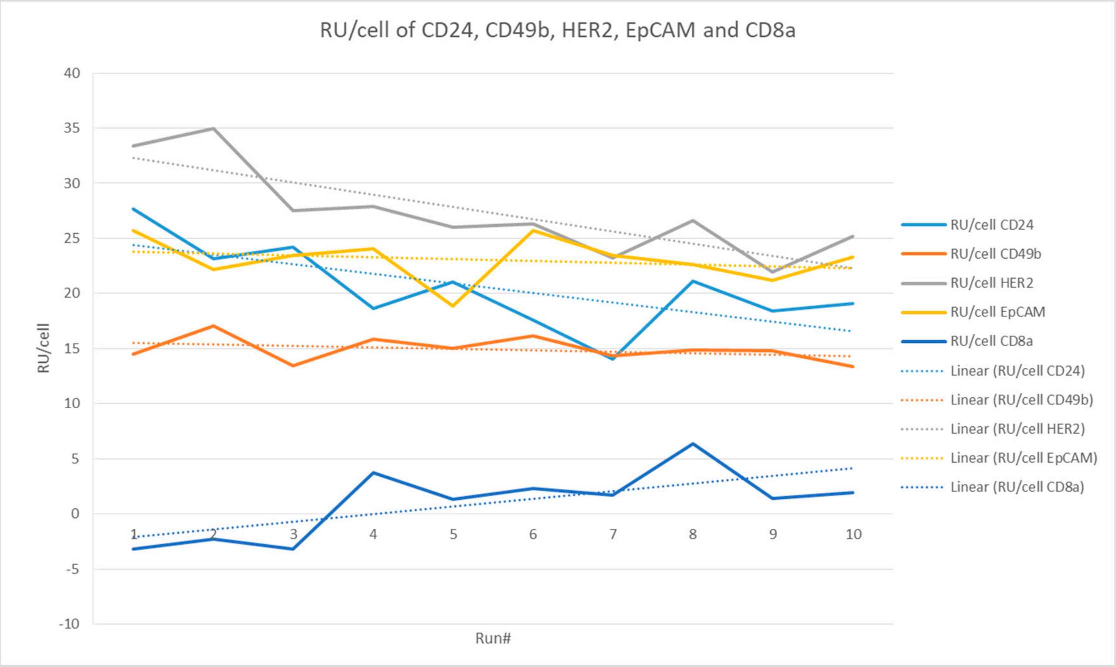

Figure S2. Graph plotting the RU/cell responses of all the markers and repeat experiments performed.

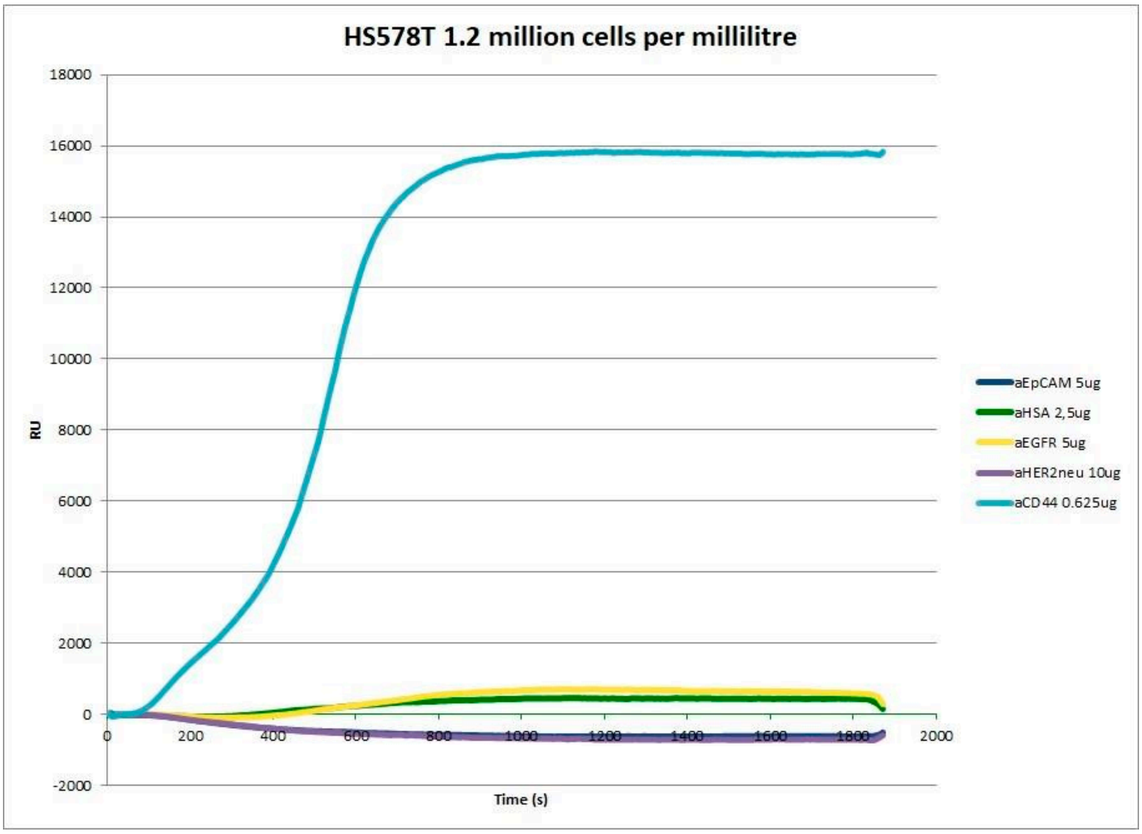

Figure 3. Sensorgram showing HS578T cells expressing a very high amount of CD44, despite the fact that the CD44 antibodies were immobilized with the lowest concentration of all the used ligands.

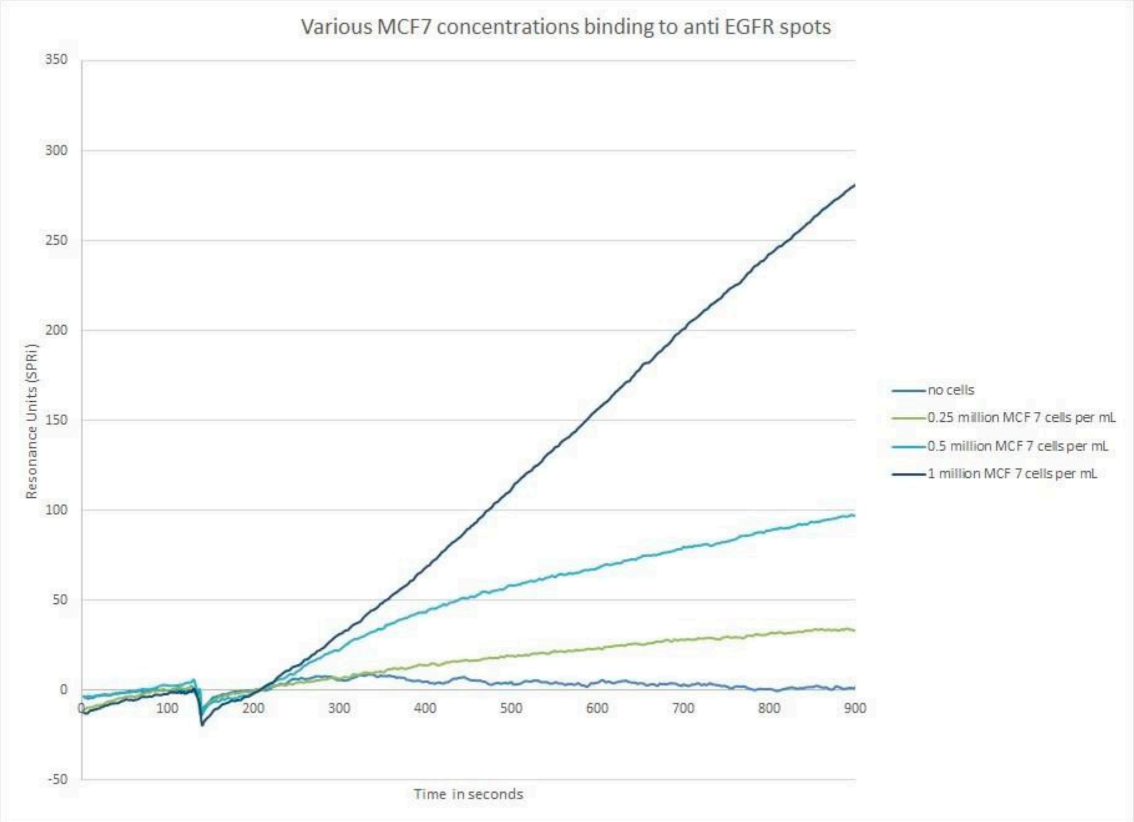

Figure S4. Overlay plot showing different amounts of MCF7 cells being analyzed for EGFR expression. As the cell concentration decreases approximately by half, so does the SPRi response.
